# Coping profiles and differences in well‐being during the COVID‐19 pandemic: A latent profile analysis

**DOI:** 10.1002/smi.3196

**Published:** 2022-09-16

**Authors:** Laura Kenntemich, Leonie von Hülsen, Ingo Schäfer, Maria Böttche, Annett Lotzin

**Affiliations:** ^1^ Department of Psychiatry and Psychotherapy University Medical Center Hamburg Eppendorf Hamburg Germany; ^2^ Department of Psychology MSH Medical School Hamburg Hamburg Germany; ^3^ Forschungsabteilung Zentrum ÜBERLEBEN Berlin Germany; ^4^ Division of Clinical Psychological Intervention Freie Universität Berlin Berlin Germany

**Keywords:** coping profiles, coronavirus, COVID‐19, disaster, mental health, pandemic, well‐being

## Abstract

During the current COVID‐19 pandemic, people need to cope with multiple stressors which may affect their well‐being. This study aimed (1) to identify latent coping profiles in the German general population, and (2) to investigate differences between these profiles in well‐being. In total, *N* = 2326 German participants were recruited as part of the European Society of Traumatic Stress Studies (ESTSS) ADJUST study from June to September 2020 using an online survey. Coping strategies were assessed using the Brief‐COPE and the Pandemic Coping Scale; well‐being was assessed using the WHO‐5 Well‐Being Index. Coping profiles were identified using latent profile analysis; differences between profiles were examined using the automatic BCH method and multiple group analyses. Five coping profiles were identified that included different types and numbers of coping strategies: (1) High functional coping (17.84%), (2) Moderate functional coping (40.63%), (3) High functional and religious coping (9.07%), (4) Low functional coping (22.06%), (5) Moderate functional and dysfunctional coping (10.40%). The identified profiles significantly differed in well‐being (*χ* ^*2*^ = 503.68, *p* <0.001). Coping profiles indicating high functional coping were associated with greater well‐being compared to coping profiles indicating low (*χ* ^*2*^ = 82.21, *p* <0.001) or primarily dysfunctional (*χ* ^*2*^ = 354.33, *p* <0.001) coping. These results provide insight into how people differ in their coping strategies when dealing with stressors in an early phase of the COVID‐19 pandemic. The study indicates higher levels of well‐being in coping profiles with more frequent use of functional strategies. To promote well‐being in the general population, it might be beneficial to train functional coping strategies in appropriate interventions that are associated with increased well‐being.

## 1 INTRODUCTION

The population worldwide is exposed to multiple stressors during the current COVID‐19 pandemic. Stressors can be defined as any ‘social and physical environmental circumstances that challenge the adaptive capabilities and resources of an organism’ (Monroe & Slavich, 2016). During the COVID‐19 pandemic, people face a wide range of stressors related to a (threat of) COVID‐19 infection and the temporary governmental restrictions to contain the spread of the virus (IMF, 2021). Many people deal with the symptoms and consequences of COVID‐19 disease. They are afraid of infecting themselves or others, are coping with the loss of loved ones, or are in self‐quarantine (Brooks et al., 2020). People are in social isolation because of contact restrictions or experienced job loss or short‐time work due to the closing of daily businesses. Many parents have to balance work and childcare because educational institutions have been temporarily closed.

International research has shown that these stressors might lead to reduced levels of well‐being within the population (e.g., South Korea: Kim et al., 2021; New Zealand: Sibley et al., 2020). German studies (Jung et al., 2020; Rek et al., 2021) found an average of 20% lower well‐being scores compared to a pre‐COVID‐19 norming sample (Brähler et al., 2007).

Well‐being is often defined as a positive affective state that is required for optimal functioning in an individual and social context (Tennant et al., 2007; Winefield et al., 2012). In a state of well‐being, individuals can cope with everyday stressors (WHO, 1948). Different researchers describe well‐being as a multidimensional construct, without a consistent definition or operationalisation (e.g., Halleröd & Seldén, 2013). The concept of well‐being stems from the assumption that mental health cannot be explained by the absence of symptoms alone (WHO, 1948). Instead, the WHO (1948) defined mental health as a state of physical, mental, and social well‐being. In conjunction with this, several studies indicate that the absence of symptoms (e.g., depressive or anxiety symptoms) is only moderately related to well‐being (Lamers et al., 2015; Trompetter et al., 2017). Keyes' (2005) two continuum model postulates that mental health and mental disorder (i.e., clinically significant impairment of a person's cognition, emotional regulation, or behaviour; American Psychiatric Association, 2013) should not be considered as two opposites along a continuum. Nevertheless, studies suggest that the assessment of well‐being has shown to be suitable for the screening and early detection of mental disorders (e.g., depression) and is better accepted by individuals than the explicit questioning of depressive symptoms (de Souza & Hidalgo, 2012). Several cross‐sectional studies have consistently shown an association between low well‐being and depression or other mental disorders (Malone & Wachholtz, 2018; Wersebe et al., 2018). First longitudinal studies showed that low well‐being could predict the risk of developing a mental disorder (Grant et al., 2013; Koivumaa‐Honkanen et al., 2004; Wood & Joseph, 2010). Thus, the assessment of well‐being could make an important contribution to the early detection and prevention of mental disorders during and in the aftermath of the COVID‐19 pandemic.

To cope with (multiple) stressors, people use different strategies to protect their well‐being (Skinner & Zimmer‐Gembeck, 2007). Previous studies suggest that people's coping style can moderate the relationship between stressors and mental health (Taylor & Stanton, 2007). Based on the Transactional Stress Model, Lazarus and Folkman (1984) define coping as a person's cognitive and behavioural efforts to manage internal and external demands during a concrete stressful situation. Over time, the conceptualisation of coping has developed from a trait‐oriented to a process‐oriented approach.

A coping response can depend on various factors, for example, the type, assessment, and context of the stressor (Dubow & Rubinlicht, 2011). There are many approaches in the literature to classify coping strategies (Skinner & Zimmer‐Gembeck, 2007). The most commonly used classifications distinguish different types of coping strategies by the copings' function (problem‐ vs. emotion‐focussed: Carver et al., 1989), or by empirical evidence on associations with mental health outcomes (functional vs. dysfunctional: Penley et al., 2002). Problem‐focussed coping strategies aim to actively change the stressor‐related problems, for example, planning or seeking instrumental support. Emotion‐focussed coping strategies aim to reduce unpleasant feelings, for example, through positive reinterpretation or acceptance (Carver et al., 1989). Furthermore, coping strategies can be differentiated by being dysfunctional versus functional depending on their impact on mental health. In this framework, behavioural disengagement, self‐blame, and substance use have been shown to be dysfunctional coping strategies (Aldao & Nolen‐Hoeksema, 2010), as they have been related to lower well‐being (Diong & Bishop, 1999), depression, or anxiety (Mahmoud et al., 2012; Xu et al., 2013). Active problem‐solving, acceptance, and daily structure are functional coping strategies and were associated with greater well‐being (Diong & Bishop, 1999) and improved mental health (Mayordomo et al., 2016; Viana Machado et al., 2020).

Associations between single coping strategies and mental health or well‐being have been investigated by international studies during an early phase of the COVID‐19 pandemic. These studies suggest that dysfunctional coping strategies (e.g., self‐blame, substance use) are associated with lower well‐being (e.g., Budimir et al., 2021; Shamblaw et al., 2021) and higher depression, anxiety, and stress symptoms (e.g., Chee et al., 2020; Mariani et al., 2020). Contrary, functional emotion‐focussed coping strategies (e.g., positive reinterpretation, humour) were associated with greater well‐being (Budimir et al., 2021; Shamblaw et al., 2021) and lower depression, anxiety, and stress symptoms (Shamblaw et al., 2021). In a German study (Saalwirth & Leipold, 2021), positive associations between problem‐focussed coping strategies and well‐being were found. Similar results were reported from an international study with 12 participating countries (Kirby et al., 2021): Problem‐focussed coping strategies, especially active problem‐solving, showed positive associations with well‐being.

A shortcoming of the conducted studies can be seen in the analysis of single coping strategies by using variable‐centred approaches. Such approaches explain relationships between specific variables among populations (Howard & Hoffman, 2018). However, these studies disregard the interplay between different coping strategies and the impact on well‐being. Ford et al. (2019) pointed out the concept of polyregulation, that is, the simultaneous or successive use of multiple coping strategies to cope with stressors. The authors suggested that this approach better accounts for how people deal with stressors. Research on polyregulation (e.g., when it is used and by whom) is limited. Lischetzke et al. (2021) examined daily coping patterns and individual differences in coping repertoires during the COVID‐19 pandemic. They identified several daily coping patterns which supports the assumption that people use polyregulation during the current pandemic. Furthermore, the study reported that people being more affected by the COVID‐19 pandemic tend to engage in polyregulation more frequently. Lischetzke et al. (2021) concluded that the need to cope and use multiple coping strategies might be particularly high during pandemic versus non‐pandemic times (Lischetzke et al., 2021). Thus, there is a need for research to consider polyregulation during the COVID‐19 pandemic and further examine the impact on well‐being.

A growing number of studies in different research fields have started to apply person‐centred approaches, for example, latent class (LCA) or profile analyses (LPA; Howard & Hoffman, 2018). These techniques can uncover underlying homogenous groups within a given sample. Applying a person‐centred approach to research on coping strategies could identify latent coping profiles that differ from each other in terms of individual coping patterns.

To date, person‐centred approaches examining individual coping profiles are limited. A few studies stemming from the time before the COVID‐19 pandemic investigated coping profiles among geriatric caregivers (Lin & Wu, 2014), family caregivers (Yuan et al., 2020), and HIV patients (Rzeszutek et al., 2017). Only a few studies examined latent coping profiles during the COVID‐19 pandemic. Pété et al. (2021) investigated coping strategies with the Brief‐COPE (Carver, 1997) in French athletes during the COVID‐19 pandemic. Four coping profiles were identified: (1) *Self‐reliant copers* with moderate levels of functional emotion‐focussed coping (positive reframing, acceptance, humour) and some rather dysfunctional coping (venting, distraction); (2) *Engaged copers* with high levels of problem‐focussed coping (planning, active coping) and moderate rather dysfunctional coping (distraction); (3) *Avoidant copers* with higher levels of dysfunctional coping (distraction, behavioural disengagement, self‐blame, denial, substance use) and moderate levels of problem‐focussed coping (cognitive restructuring, problem‐solving); (4) *Active & Social copers* with high levels of problem‐focussed (cognitive restructuring, problem‐solving) and dysfunctional coping (distraction) as well as moderate levels of emotion‐focussed coping (support seeking, instrumental support, emotional support, religion). The profile of *Avoidant copers* was related to greater anxiety symptoms and higher levels of perceived threat of the pandemic compared to the other profiles. Differences in well‐being between the profiles were not examined. Kavčič et al. (2021) identified three coping profiles (*Engaged, Low, Avoidant coping*) based on the Brief‐COPE (Carver, 1997) in the Slovenian population during the COVID‐19 pandemic. While the *Engaged coping* profile (i.e., high levels of functional problem‐ and emotion‐focussed coping) was associated with the highest level of well‐being, the *Disengaged* profile (i.e., low levels of functional problem‐ and emotion‐focussed strategies, high levels of dysfunctional coping such as behavioural disengagement, denial) and *Avoidant coping* profile (i.e., high levels of dysfunctional strategies such as self‐blame, substance use) were associated with the highest levels of anxiety and distress. The lowest levels of well‐being were reported in the *Disengaged* profile.

To sum up, these studies found similar profiles of individuals combining problem‐focussed, emotion‐focussed, and dysfunctional coping strategies in an individual pattern during the COVID‐19 pandemic. In line with Pété et al. (2021) and Kavčič et al. (2021), we assessed coping with the Brief‐COPE questionnaire (Carver, 1997). The questionnaire covers a wide range of different coping strategies. The Brief‐COPE is based on different theoretical approaches, such as the transactional model of stress and coping (Lazarus & Folkman, 1984) or the model of behavioural self‐regulation (Carver & Scheier, 1981). According to Cooper et al. (2008), the items of the Brief‐COPE can be divided into three domains: Active coping, planning, and instrumental support as problem‐focussed coping; emotional support, positive reinterpretation, acceptance, humour, and religion as emotion‐focussed coping; distraction, venting, self‐blame, behavioural disengagement, denial, and substance use as dysfunctional coping. However, the Brief‐COPE was developed to measure coping with everyday stressors, not pandemic‐specific stressors. As coping strategies can differ depending on the circumstances, it is likely that there are specific strategies for dealing with stressors during a pandemic. Several researchers pointed out the need to assess coping that is unique for a pandemic (e.g., Rahman et al., 2020). Thus, we measured pandemic‐related coping with a self‐constructed scale (Pandemic Coping Scale [PCS]; Lotzin et al., 2021). The PCS was constructed based on currently published recommendations for managing the COVID‐19 pandemic (for details, see Lotzin et al., 2021) and is intended to be an addition to the Brief‐COPE. For example, the PCS captures preventive coping (e.g., following governmental recommendations) or maintaining daily routines (despite lockdown and working from home).

Given the multiple stressors during the current pandemic and first evidence indicating a general reduction of well‐being (Jung et al., 2020; Rek et al., 2021), the present study aimed to (1) identify latent coping profiles in the German general population during the first year of the COVID‐19 pandemic and to (2) investigate differences between these profiles in well‐being.

Even though latent profile analysis is an explorative approach, we have formulated hypotheses based on previous studies that used a person‐centred approach during the COVID‐19 pandemic (Kavčič et al., 2021; Pété et al., 2021). First, we hypothesized to identify three to four latent coping profiles in the German general population during the COVID‐19 pandemic (H_1_). Furthermore, we expected to find latent profiles with predominantly (1) functional problem‐ and/or emotion‐focussed coping (e.g., planning, acceptance), (2) dysfunctional coping (e.g., venting, distraction), and (3) less use of coping in general (H_2_). In line with empirical findings (Kavčič et al., 2021), we assumed that people with frequent use of problem‐ and emotion‐focussed coping have significantly higher levels of well‐being than people with frequent use of dysfunctional coping or general low use of coping (H_3_).

A better understanding of differences between coping profiles in well‐being could make a relevant contribution to the prevention of mental disorders and the development of specific training programs for pandemics or other disasters.

## 2 METHODS

### 2.1 Study design and setting

The participants were recruited as part of a pan‐European cohort study (‘ADJUST study’) of the European Society for Traumatic Stress Studies (ESTSS). The ADJUST study longitudinally investigates risk and protective factors, stressors, coping, and symptoms of an adjustment disorder during the COVID‐19 pandemic in 11 predominantly European countries (Lotzin et al., 2020).

### 2.2 Procedure

Data from the German general population were collected between June and September 2020. The survey was actively advertised via social platforms (e.g., Facebook, Twitter), leisure and interest groups (e.g., bicycle or car clubs), newsletters or organisations (e.g., newsletters of large companies), and via advertisements in newspapers and magazines. Study information was also disseminated through universities, different stakeholders, and professional organisations. Interested individuals received an invitation link to the online platform *Limesurvey* (LimeSurvey GmbH, Version 3.22). After providing consent, they could complete an online survey.

### 2.3 Participants

Data for this study were drawn from the first wave of the data assessment. Data from *N* = 2326 participants from the German general population were used for the present study. Inclusion criteria were (1) at least 18 years of age, (2) ability to read and write in German, (3) willingness to participate in the study, and (4) at least one completed item on coping strategies. As this study is a secondary data analysis drawn from a larger study, no a‐priori sample size calculation was conducted for this study. A post‐hoc power analysis (*α* = 0.05; *R* ^*2*^ = 0.164; *N* = 2236) using G*Power Version 3.1.9.2 (Faul et al., 2007) indicated an adequate power of 100% for the analyses of the present study.

### 2.4 Measures

#### 2.4.1 Coping

Coping was assessed using the Brief‐COPE (Carver, 1997; Knoll et al., 2005). The Brief‐COPE is the short version of the Coping Orientation to Problems Experienced (COPE; Carver et al., 1989) inventory. The Brief‐COPE measures 14 coping strategies by 28 items on a four‐point Likert scale (0 = ‘I have not been doing this at all’; 1 = ‘I've been doing this a little bit’; 2 = ‘I've been doing this a medium amount’; 3 = ‘I've been doing this a lot’). The coping strategies can be divided into three domains (Cooper et al., 2008): Problem‐focussed coping, emotion‐focussed coping, and dysfunctional coping. Overall, these domains showed good internal consistencies (problem‐focussed: *α* = 0.84; emotion‐focussed: *α* = 0.72; dysfunctional: *α* = 0.75).

Pandemic‐specific coping strategies were assessed by a self‐constructed 13‐item questionnaire (PCS; Lotzin et al., 2021). The items were rated on a four‐point scale, equivalent to the Brief‐COPE. To investigate the factor structure of the pandemic‐specific coping strategies, an exploratory factor analysis (EFA) was conducted among the German sample of the ADJUST study (for details, see Lotzin et al., 2021). The EFA suggested a four‐factor solution with factor loadings accounting for 44.6% of the total variance (Factor 1: Healthy lifestyle, Factor 2: Enjoyable activities, Factor 3: Daily structure, Factor 4: Preventive measures). The PCS showed adequate factorial validity and reliability for the four measured dimensions of coping except for ‘Preventive measures’ to assess coping during the COVID‐19 pandemic.

#### 2.4.2 Well‐being

Well‐being was assessed using the World Health Organisation's Well‐Being Index (WHO‐5 Well‐Being Index; Bech et al., 2003), a commonly used instrument to assess subjective well‐being. The WHO‐5 is a global 5‐item self‐report measure (Topp et al., 2015) and is available in two different versions; version II was used in this study, for which advantageous psychometric properties have been reported (Brähler et al., 2007). The items were rated on a five‐point scale (0 = ‘At no time’; 1 = ‘Some of the time’; 2 = ‘Less than half of the time’; 3 = ‘More than half of the time’; 4 = ‘Most of the time’; 5 = ‘All the time’), referring to the last 2 weeks. An index value (*Min* = 0, *Max* = 25) can be generated with higher values reflecting a higher well‐being. Values below ‘13’ indicate low well‐being and can be interpreted as an indicator for further diagnosis of a depressive disorder (Brähler et al., 2007). It is recommended to convert the raw index score into a percentage scale from 0 (*Min*) to 100 (*Max*) (Topp et al., 2015). Using a WHO‐5 cut‐off score of ≤50 is recommended to screen for clinical depression (Topp et al., 2015). The WHO‐5 version II showed good internal consistencies (Cronbach's *α* = 0.92; Guttmann's test half‐reliability *r*_*tt*_ = 0.87).

#### 2.4.3 Data analysis

In a first step, a latent profile analysis (Lazarsfeld & Henry, 1968) was conducted with the statistical programme *MPlus* (version 7.13, macOS) to identify latent coping profiles based on the 18 coping scales measured by the Brief‐COPE and PCS (Brief‐COPE: Carver, 1997; Knoll et al., 2005; PCS: Lotzin et al., 2021). A LPA is a person‐centred statistical method that aims to divide individuals into latent, homogeneous classes (or profiles) based on their response behaviour to the variables of interest. Model parameters were estimated using maximum likelihood estimation and robust standard errors. Complete data were available for 99.98% of the participants. To avoid local maxima, 1000 random sets of initial values were used in the first step, 100 random sets in the second step of the optimization, and 50 iterations in the initial phase.

To determine the optimal number of profiles, the Bayesian Information Criterion (BIC; Schwarz, 1978), the sample size adjusted BIC (ssBIC; Sclove, 1987), the Lo‐Mendell‐Rubin adjusted Likelihood difference test (LMR test; Lo et al., 2001), and the Bootstrap Likelihood Ratio difference test (BLR test; McLachlan & Peel, 2000) were used as model fit criteria to compare the different models with different class solutions. Nylund et al. (2007) suggest that these goodness‐of‐fit criteria are the best for determining the optimal class size and classification goodness. The information criteria BIC and ssBIC were used to compare the goodness‐of‐fit of the competing models, preferring the model with the lowest BIC value. The BIC is based on a log‐likelihood function and a penalty term for model complexity to avoid an over‐fitted solution. The LMR and BLR tests were used to compare models with increasing numbers of classes to examine which class solution can best represent the data structure. A *p*‐value ≤ 0.05 indicates that the estimated model represents the data structure significantly better than a model with k‐1 classes (Nylund et al., 2007). Entropy (Ramaswamy et al., 1993) was used as a standardized measure of the accuracy of class assignments. An entropy ≥0.80 indicates an acceptable correct assignment probability (Muthén, 2004). Additionally, the interpretability and parsimony of classes were considered as well as a large total sample (*N* > 500; Nylund et al., 2007) and sufficiently large profile sizes (*n* > 25 or *n* > 1% of the total sample; Lubke & Neale, 2006). As a further measure of the goodness of the classifications, the estimated mean class assignment probabilities are supposed to be > 0.80 (Nylund et al., 2007).

After selecting the optimal latent profile model, we conducted multiple group analyses to examine differences in well‐being between the coping profiles. Therefore, we have used the automatic BCH (Bolck et al., 2004) approach in *MPlus*. This procedure uses Wald tests to compare the mean scores of a continuous or categorical distal outcome (i.e., well‐being) among different groups (i.e., coping profiles). Several simulation studies suggest that this provides robust results, also for non‐normal distributed variables (Asparouhov & Muthén, 2021; Bakk & Vermunt, 2016). The BCH procedure considers individual uncertainties in profile classification by using observation weights that reflect measurement errors in the latent profile variable (Asparouhov & Muthén, 2021). All *p‐*values were corrected with the Bonferroni‐Holm method (Holm, 1979) to take into account multiple group testing. A significance level of *α* = 0.05 was set for all calculations.

## 3 RESULTS

### 3.1 Sample characteristics

The study included *n* = 2326 German participants aged 18–82 years (*M* = 41.00, *SD* = 12.46; Table 1). The sample can be characterized as predominantly female, with high levels of education and income (Table 1). Only 0.73% (*n* = 17) of the participants had already been infected with the coronavirus, all of whom had recovered by the time of the survey. One‐fifth of the sample (*n* = 501; 21.54%) considered themselves at risk for a severe or life‐threatening course of COVID‐19 disease. Pandemic‐specific coping strategies (PCS, Table 2) were frequently used, especially the ‘Preventive measures’ (*M* = 2.70, *SD* = 0.48). Among the general coping strategies (Brief‐COPE, Table 2), the emotion‐focussed strategies ‘Acceptance’ (*M* = 1.78, *SD* = 0.85) and ‘Positive reinterpretation’ (*M* = 1.59, *SD* = 0.90), as well as the dysfunctional strategy ‘Self‐distraction’ (*M* = 1.61, *SD* = 0.81), were used most often. The mean percentage well‐being score (WHO‐5, Table 2) of the sample was at a medium level (*M* = 51.30, *SD* = 22.16, *Min*: 0, *Max*: 100).

**TABLE 1:** Sociodemographic characteristics (*N* = 2326)

Characteristics	
Age (years)	*M (SD)*
Mean	41.00 (12.46)
Range	18–82
Gender	*n* (%)
Male	682 (29.32)
Female	1634 (70.25)
Diverse	10 (0.43)
Education	
<10 years of schooling	7 (0.30)
≥10 years of schooling	289 (12.42)
Vocational studies	818 (35.17)
Completed studies	1212 (52.11)
Income^a^	
Very low (<500 €)	80 (3.59)
Low (500 < 1000 €)	145 (6.50)
Medium (1000 < 3000 €)	901 (40.40)
High (≥3000 €)	1104 (49.51)
COVID‐19 infection (tested positive)	
Yes, recovered	17 (0.73)
No	2309 (99.27)
At risk for severe COVID‐19 infection	
Yes	501 (21.54)
No	1825 (78.46)

^a^ *N* = 2230.

**TABLE 2:** Coping strategies and clinical characteristics (*N* = 2326)

Characteristics	
Brief‐COPE	*M (SD)*
Active coping^a^	1.38 (0.81)
Planning^b^	1.58 (0.82)
Instrumental support^b^	0.89 (0.80)
Positive reinterpretation^c^	1.59 (0.90)
Acceptance^b^	1.78 (0.85)
Humour^b^	1.14 (0.85)
Religion^b^	0.37 (0.71)
Emotional support^c^	1.28 (0.86)
Self‐distraction^c^	1.61 (0.81)
Denial^d^	0.29 (0.55)
Venting^c^	0.94 (0.74)
Substance use^c^	0.36 (0.68)
Behavioural disengagement^c^	0.49 (0.60)
Self‐blame^b^	0.35 (0.63)
Pandemic Coping Scale	
Healthy lifestyle	1.59 (0.73)
Enjoyable activities^e^	1.89 (0.64)
Daily structure^e^	1.91 (0.91)
Preventive measures	2.70 (0.48)
WHO‐5 Well‐Being Index^f^	51.30 (22.16)

*Note*: Brief‐COPE, Pandemic Coping Scale: (0 = ‘I have not been doing this at all’; 1 = ‘I've been doing this a little bit’; 2 = ‘I've been doing this a medium amount’; 3 = ‘I've been doing this a lot’). WHO‐5 Well‐Being Index (0%–100%).

^a^ *n* = 2323.

^b^ *n* = 2320.

^c^ *n* = 2321.

^d^ *n* = 2322.

^e^ *n* = 2325.

^f^ *n* = 2271.

### 3.2 Coping profiles during the COVID‐19 pandemic

The statistical model fit indices for a 2‐ to 7‐class solution are shown in Table 3. The BIC and ssBIC values declined with an increasing number of classes continuously until the 6‐class solution. The 7‐class solution showed a higher BIC and ssBIC value than the 6‐class solution, indicating a lower goodness‐of‐fit for the 7‐class solution. The entropy showed a value ≥ 0.8 for all models, suggesting good classification goodness between the different classes (Muthén, 2004). The LMR test showed a significantly better representation of the data structure using a 5‐class solution compared to a 4‐class solution (*p* < 0.001). No significant difference was found between the 5‐ and the 6‐class solution (*p* = 0.484, Table 3). However, the BLR test also showed a significant difference for the 6‐ compared to the 5‐class solution (*p* < 0.001).

**TABLE 3:** Model fit indices of latent coping profiles during the COVID‐19 pandemic

Class	Log likeli‐hood	BIC	ssBIC	LMR	LMR test *p*‐value	BLR	BLR test *p*‐value	Entropy
2	−43,937.6	88,301.6	88,126.8	−46,192.6	<0.001	−46,192.6	<0.001	0.80
3	−43,164.5	86,902.7	86,667.6	−43,937.6	<0.001	−43,937.6	<0.001	0.86
4	−42,605.7	85,932.4	85,636.9	−43,164.5	<0.001	−43,164.5	<0.001	0.81
**5**	**−42,136.7**	**85,141.6**	**84,785.7**	**−42,605.7**	**<0.001**	**−42,605.7**	**<0.001**	**0.84**
6	−41,720.4	84,456.2	84,040.0	−42,149.2	0.484	−42,149.2	<0.001	0.86
7	−41,462.7	84,840.7	84,424.5	−42,149.2	0.240	−42,149.2	<0.001	0.85

*Note*: Model fit indices for the favoured model are in bold.

Abbreviations: BIC, Bayesian Information Criterion; BLR, Bootstrapped Likelihood Ratio; LMR, Lo‐Mendell‐Rubin adjusted Likelihood Ratio; ssBIC, sample size adjusted Bayesian Information Criterion.

**FIGURE 1:**
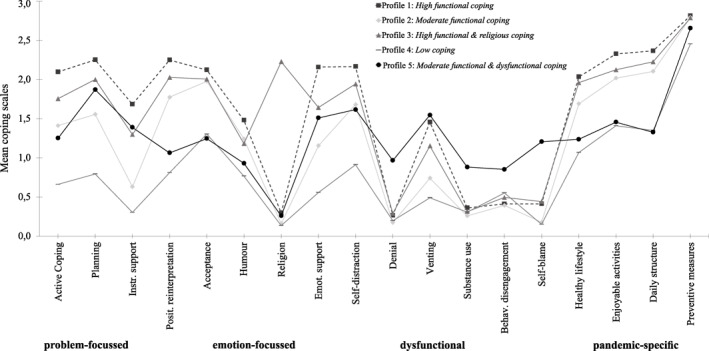
Profiles of coping behaviour during the COVID‐19 pandemic in the German general population. Coping scales = Brief‐COPE, Pandemic Coping Scales: (0 = ‘I have not been doing this at all’; 1 = ‘I've been doing this a little bit’; 2 = ‘I've been doing this a medium amount’; 3 = ‘I've been doing this a lot’). Instr. support, Instrumental support; Posit. reinterpretation, Positive reinterpretation; Emot. support, Emotional support; Behav. disengagement, Behavioural disengagement.

Compared to the 4‐class solution, the 5‐class solution contained an additional profile with high values in the coping strategy ‘Religion’. Compared to the 5‐class solution, the 6‐class solution contained an additional profile with high scores in the dysfunctional coping strategy ‘Self‐blame’. However, this profile accounted for only a small proportion (*n* = 159, 6.8%) of the total sample. In the 5‐class solution, participants with high scores on dysfunctional strategies were combined into one profile (*n* = 242; 10.4%). Considering the statistical information measures, entropy, parsimony as well as interpretability of the classes, the 5‐class solution is to be preferred. The profile sizes for the 5‐class solution were sufficiently large (all *n* > 200; Lubke & Neale, 2006) and the mean class assignment probabilities for all profiles were satisfactory (>0.80; Nylund et al., 2007). In terms of content, the 5‐class solution included informative profiles that differed in the frequency of coping and the coping strategies used (Figure 1).

Profile 1 (*n* = 415; 17.84%) was representative of individuals with high scores in functional problem‐ and emotion‐focussed as well as pandemic‐specific coping strategies, and the dysfunctional coping strategy ‘Self‐distraction’. Moderate scores were found for the dysfunctional coping strategy ‘Venting’. This profile was labelled *High functional coping*.

Profile 2 (*n* = 945; 40.63%) characterized individuals with moderate levels of functional problem‐ and emotion‐focussed and pandemic‐specific coping strategies. Moderate scores were found for the dysfunctional coping strategy ‘Self‐distraction’. This profile was labelled *Moderate functional coping*.

Profile 3 (*n* = 211; 9.07%) clustered individuals with high levels of functional problem‐ and emotion‐focussed coping strategies. This profile differed from the remaining profiles in the more frequent use of the emotion‐focussed coping strategy ‘Religion’. The dysfunctional coping strategy ‘Self‐distraction’ and the functional pandemic‐specific coping strategies were also frequently used. Therefore, this profile was labelled *High functional and religious coping*.

Profile 4 (*n* = 513; 22.06%) was primarily composed of individuals with low frequencies of using any of the coping domains. Only pandemic‐specific coping strategies (e.g., preventive measures) and the functional emotion‐focussed coping strategy ‘Acceptance’ were rarely to moderately frequently used. Therefore, Profile 4 was labelled *Low coping*.

Profile 5 (*n* = 242; 10.40%) described individuals with moderate levels of functional problem‐focussed and dysfunctional (e.g., distraction, venting) coping strategies. Low to moderate levels were found in functional emotion‐focussed and pandemic‐specific coping. High scores were only found for the use of ‘Preventive measures’. Compared to all other profiles, individuals in Profile 5 used dysfunctional strategies (e.g., denial, substance use, self‐blame) more often. Therefore, this profile was labelled *Moderate functional and dysfunctional coping*.

The estimated mean scores of the coping strategies for the latent coping profiles are shown in the supplements (Table S1).

### 3.3 Differences between the coping profiles in well‐being during the COVID‐19 pandemic

Descriptively, Profile 1 (*High functional coping*) showed the highest well‐being score on average (*M* = 59.09, *SE* = 1.10; Table 4), followed by Profile 2 (*Moderate functional coping: M* = 57.04, *SE* = 0.76) and Profile 3 (*High functional and religious coping: M* = 54.69, *SE* = 1.61). Profile 4 (*Low coping*: *M* = 44.86, *SE* = 1.14) and Profile 5 (*Moderate functional and dysfunctional coping: M* = 26.82, *SE* = 1.28) had the lowest average well‐being scores. Multiple group analyses revealed statistically significant differences in well‐being among the coping profiles (*χ* ^*2*^ = 503.68, *p* <0.001, Table S2). These differences exist between Profiles 1 and 4 (*χ* ^*2*^ = 82.21, *p* <0.001), Profiles 1 and 5 (*χ* ^*2*^ = 354.33, *p* <0.001), Profiles 2 and 4 (*χ* ^*2*^ = 70.30, *p* <0.001), Profiles 2 and 5 (*χ* ^*2*^ = 397.71, *p* <0.001), Profiles 3 and 4 (*χ* ^*2*^ = 24.67, *p* <0.001), Profiles 3 and 5 (*χ* ^*2*^ = 181.57, *p* <0.001) and Profiles 4 and 5 (*χ* ^*2*^ = 106.07, *p*  <0.001).

**TABLE 4:** Multiple group analyses of the differences between the coping profiles in well‐being using the BCH approach

Variable: Well‐being (WHO‐5 total score)	*M*	*SE*	Differences between profiles
Profile 1: *High functional coping*	59.09	1.10	1 = 2 = 3 > 4 > 5
Profile 2: *Moderate functional coping*	57.04	0.76	
Profile 3: *High functional & religious coping*	54.69	1.61	
Profile 4: *Low coping*	44.86	1.14	
Profile 5: *Moderate functional & dysfunctional coping*	26.82	1.28	

*Note*: Well‐being: WHO‐5 Well‐Being Index (0%–100%). Differences were analysed using Wald tests and Bonferroni‐Holm correction.

## 4 DISCUSSION

The COVID‐19 pandemic is associated with multiple stressors that threaten the well‐being and mental health of the general population. As the pandemic proceeds, it becomes increasingly important to gain a better understanding of protective coping strategies during the pandemic for well‐being. Therefore, this study aimed to identify latent coping profiles among adults in the German general population during the COVID‐19 pandemic in summer and autumn 2020 and to examine differences between these profiles in well‐being.

### 4.1 Latent coping profiles during the COVID‐19 pandemic

We identified five latent coping profiles in the German general population during an early phase of the COVID‐19 pandemic. This is one more profile than previously expected (H_1_) and as found by prior studies (Kavčič et al., 2021; Pété et al., 2021) during the pandemic. Since we additionally assessed pandemic‐related coping, it is plausible that we could distinguish more profiles.

Two out of 10 people (17.84%, Profile 1) showed *High functional coping* so they used coping strategies that are defined as functional in the literature (e.g., acceptance, planning, daily structure). Nakamura and Orth (2005) suggested that ‘Acceptance’ could be a functional response to unchangeable situations with low personal control. Active acceptance of an unchangeable situation could be of great functional importance for well‐being, especially during the COVID‐19 pandemic, which comprises many unpredictable and unchangeable circumstances (Eisenbeck et al., 2021). However, Profile 1 also showed moderate to high levels of ‘Self‐distraction’ and ‘Venting’, often classified as dysfunctional in the literature. Wolgast and Lundh (2017) pointed out that ‘Self‐distraction’ can be either functional or dysfunctional, depending on whether it is combined with ‘Acceptance’ or avoidant coping strategies. Therefore, the functionality of the coping strategy ‘Self‐distraction’ may depend on whether it is primarily used to (1) avoid aversive feelings or (2) shift attention in the short term, with a willingness to address the avoided feelings later (Wolgast & Lundh, 2017).

Previous studies have labelled profiles with a similar coping pattern as *Hybrid* (Lin & Wu, 2014), *Mixed* (Rzeszutek et al., 2017), *High* (Yuan et al., 2020), *Engaged* (Kavčič et al., 2021; Pété et al., 2021), or *Active and Social* (Pété et al., 2021) coping. Pété et al. (2021) identified two different high functional coping profiles: The *Active and Social* coping profile showed higher frequencies of ‘Self‐distraction’ and ‘Support seeking’ than the *Engaged* *coping* profile. We could not find this differentiation in our study.

Four out of 10 individuals (40.63%, Profile 2), and thus the largest part of the sample, showed *Moderate functional coping*. This profile combined functional problem‐and emotion‐focussed as well as pandemic‐specific coping strategies with moderate frequency. Contrary to Profile 1, ‘Instrumental support’ was rarely used by this profile. Yuan et al. (2020) found a similar profile (*Moderate coping*) with moderate use of functional coping strategies as well as the coping strategy ‘Self‐distraction’. As discussed in conjunction with Profile 1, ‘Self‐distraction’ might be seen as functional as it is associated with well‐being (Wolgast & Lundh, 2017). The other studies did not report a similar moderate coping profile.

One in 10 individuals (9.07%, Profile 3) was characterized by a profile which we named *High functional and religious coping*. Profile 3 showed a similar coping pattern compared to Profile 1, with the exception that individuals frequently used the coping strategy ‘Religion’. Considering that this was the smallest profile, only a small proportion of the German population was using religious coping during the COVID‐19 pandemic. Individuals using ‘Religion’ as a coping strategy seem to generally use coping strategies often compared to other profiles. While some studies report a protective effect of religious coping on the negative psychological impact of the COVID‐19 pandemic (Thomas & Barbato, 2020; Zacher & Rudolph, 2021), DeRossett et al. (2021) suggest that religious coping can also be dysfunctional (e.g., if someone feels punished by God because of the pandemic). Counted et al. (2022) found a moderating effect of religious coping on the relationship between hope and well‐being during the COVID‐19 pandemic. When people reported low hope, higher well‐being was associated with a high frequency of positive religious coping; lower well‐being in turn was associated with a high frequency of negative dysfunctional coping. However, the distinction between positive and negative religious coping was not assessed in our study. No other study reported a separate high functional coping profile with high use of ‘Religion’.

Two out of 10 individuals (22.06%, Profile 4) showed *Low coping*. Individuals in this profile used all coping strategies infrequently. Thus, almost one‐fifth of the current German sample responded to the COVID‐19 pandemic with some form of passive general coping. However, COVID‐19‐specific coping strategies (e.g., preventive measures) were used frequently. Previous studies described profiles with generally low coping as *Unpatterned* (Lin & Wu, 2014), *Generally low* or *Lowest intensity* (Rzeszutek et al., 2017), *Low* (Yuan et al., 2020), or *Disengaged* (Kavčič et al., 2021) coping. Pété et al. (2021) could not identify a coping profile with general low use of coping strategies.

One in 10 individuals (10.40%, Profile 5) belonged to a profile which we named *Moderate functional and dysfunctional coping*. This profile mostly used the coping strategies ‘Planning’, ‘Emotional support’, ‘Self‐distraction’, and ‘Preventive measures’. Furthermore, Profile 5 had the highest levels of dysfunctional coping strategies (e.g., denial, substance use, self‐blame) compared to the other profiles. Kavčič et al. (2021) and Pété et al. (2021) labelled profiles with a similar coping pattern as *Avoidant coping;* Rzeszutekt et al. (2017) as *High* and *Highest intensity* coping. However, the last two showed a higher coping frequency. Lin and Wu (2014) and Yuan et al. (2020) did not report profiles with a similar coping pattern.

In summary, coping profiles in this study showed similarities to those of previous studies (e.g., Kavčič et al., 2021; Pété et al., 2021; Yuan et al., 2020). As hypothesized (H_2_), we found profiles with a predominantly functional problem‐ and/or emotion‐focussed coping (Profile 1, 2 and 3), dysfunctional coping (Profile 5), and low use of coping in general (Profile 4). However, some pre‐pandemic studies reported coping profiles that we could not find in our study. For instance, Lin and Wu (2014) identified a profile with exclusive use of emotion‐focussed coping strategies. Since we did not find such a profile, individuals might tend to combine different coping strategies during the COVID‐19 pandemic, rather than limiting themselves to certain strategies (e.g., reducing negative feelings through emotion‐focussed coping). Rzeszutek et al. (2017) found profiles with *High, Highest* and *Lowest intensity* coping. In contrast to our study, the profiles with higher intensity coping further used dysfunctional coping strategies. Additionally, we could not find profiles with very high or low coping frequencies in our study. Pété et al. (2021) reported an additional profile with *Self‐reliant coping*, which was characterized by moderate functional emotion‐focussed coping and several dysfunctional strategies (e.g., distraction, venting). This profile showed a similar coping pattern as Profile 5 of our study. However, the difference is that Profile 5 also showed a moderate frequency of problem‐focussed coping strategies.

Differences between the studies regarding latent coping profiles might exist due to several factors. First, the reported studies examined different samples. While the present study included individuals from the German general population, most of the studies consisted of specific subgroups. Second, different instruments and a different number of items were used within the studies. Furthermore, in contrast to the other studies, we assessed pandemic‐specific coping strategies. Third, the timing and context of the survey varied across the studies. Only the present study, as well as the study of Kavčič et al. (2021) and Pété et al. (2021), were conducted during the COVID‐19 pandemic. As coping strategies can vary depending on the context and the level of distress (Dubow & Rubinlicht, 2011; Fischer et al., 2021), the identification of different latent coping profiles may also be explained.

### 4.2 Differences between the coping profiles in well‐being during the COVID‐19 pandemic

We found considerable differences between the latent coping profiles in well‐being. Compared to all other profiles, individuals in the *High functional coping* profile showed the highest levels of well‐being. Thus, the frequent use of functional problem‐focussed and emotion‐focussed coping (especially planning, positive reinterpretation, and acceptance) appears to be positively associated with well‐being during the pandemic. Individuals with *Moderate functional coping* or *High functional and religious coping* showed slightly but not significantly lower levels of well‐being. So, the frequency of functional coping or the presence of religious coping does not seem to have a decisive effect on well‐being. As expected (H_3_), individuals with *Low coping* or *Moderate functional and dysfunctional coping* showed 14.2% and 32.3% lower well‐being scores than the *High functional coping* profile, respectively. Thus, the profile with *Moderate functional and dysfunctional coping* is related to the lowest well‐being. This could imply that the positive impact of functional coping on well‐being is negated by the additional use of dysfunctional coping strategies. In addition, the results indicate that low use of all coping strategies is associated with better well‐being than the combination of functional and dysfunctional strategies. This might be because individuals from the *Low Coping* profile had the highest scores for the strategies ‘Acceptance’ and ‘Self‐distraction’. Thus, the general low coping could also be a kind of short‐term avoidance or acceptance of pandemic‐related events. In turn, this could be associated with higher well‐being than coping with the pandemic by using dysfunctional strategies such as self‐blame, venting, or substance use.

The findings on differences between latent coping profiles in well‐being are consistent with several studies before and during the COVID‐19 pandemic that have reported positive associations between functional coping strategies and negative associations between dysfunctional coping strategies and well‐being (e.g., Budimir et al., 2021; McFadden et al., 2021; Zacher & Rudolph, 2021). Consistently, Kavčič et al. (2021) showed that individuals using *Engaged coping* reported the highest levels of well‐being, while individuals with *Disengaged coping* reported the lowest during the COVID‐19 pandemic. In turn, individuals with *Avoidant coping* showed the highest anxiety and stress scores. In contrast, Rzeszutek et al. (2017) found the lowest well‐being in individuals with *High functional coping*, whereas individuals with *Low coping* reported the highest levels of well‐being. Moreover, caregivers with *Moderate coping* in the study of Yuan et al. (2020) reported more severe depressive symptoms than caregivers with *Low coping*. These results may indicate that low use of coping strategies could also reflect low perceived distress, associated with higher levels of well‐being and less depressive symptoms. On the other hand, very high use of coping strategies could indicate high levels of distress, which is reflected in reduced well‐being. Future studies should investigate to what extent perceived stress influences the relationship between coping patterns and well‐being. In addition, there may be other confounding variables (e.g., gender, age, pre‐existing physical or mental illness) that influence the association and should be controlled in further studies. However, the contradictory results of the different studies could again result from the heterogeneous samples, the different assessments of well‐being, and the context of the survey.

The results of the present study suggest that the combination of functional coping strategies during the COVID‐19 pandemic is associated with higher levels of well‐being in the German population. In line with other studies from an early phase of the pandemic, this study found an average reduction in well‐being of about 20% compared to a norm sample (Brähler et al., 2007). The average level of well‐being of the sample was only marginally above the cut‐off value of 50, recommended by Topp et al. (2015) for screening depressive disorders. This secondary result is another indication that adults in the German general population are burdened by the COVID‐19 pandemic. Individuals with *Low coping* or *Moderate functional and dysfunctional coping* were on average about 5%–23% below this cut‐off value.

### 4.3 Limitations

This is one of the first German studies examining coping strategies and differences in well‐being in an early phase of the COVID‐19 pandemic. A strength of this study is the large sample size. Furthermore, well‐validated instruments (WHO‐5: Bech et al., 2003; Brief‐COPE: Carver, 1997) and questionnaires developed specifically for the COVID‐19 pandemic (PCS: Lotzin et al., 2021) were used.

A limitation of the study is the use of a non‐probabilistic sample of the German general population, overrepresenting women and individuals with high education, income, and internet access. Furthermore, the self‐selection of the participants might limit the generalizability of the results (Schaurer & Weiß, 2020). Individuals with higher psychological distress could be more likely to complete a mental health questionnaire during the COVID‐19 pandemic. This may explain the low average well‐being in the sample compared to a pre‐COVID comparison sample. Furthermore, the use of self‐report questionnaires could have contributed to systematic biases (Bowling, 2005). Another limitation of this study concerns the self‐constructed questionnaire (PCS: Lotzin et al., 2021) used in the study project to assess COVID‐19‐specific coping strategies, which was psychometrically examined but not evaluated in previous studies. Due to the cross‐sectional design, the study does not allow causal conclusions about the relationship between latent coping profiles and well‐being. Therefore, it would also be possible that well‐being has an impact on the type and frequency of coping.

Furthermore, the *High functional coping* profile only showed high use of functional coping strategies compared to the other profiles. Descriptively, individuals have used functional strategies only sometimes to often. Similarly, the comparatively high well‐being of this profile was still about 10% lower compared to a pre‐COVID‐19 comparison sample.

Pre‐existing (mental) health disorders or high levels of distress that might overlap with the dependent variable were not considered. Furthermore, the data were collected in summer and autumn 2020. This timing may also affect the results, as infection rates and constraints in Germany were lower during this period than during the acute phases of the lockdown (IMF, 2021).

Another limitation can be seen in the use of an exploratory statistical technique (LPA) to identify latent homogeneous groups within the sample. Furthermore, not all statistical model fit indices indicated that a 5‐class solution best represents the data. However, we used the automatic BCH method to consider inaccuracies in profile classifications when examining differences in well‐being between the coping profiles. Further studies should examine whether a 5‐class solution best describes coping profiles during the COVID‐19 pandemic in the German general population.

## 5 CONCLUSION

This study provides an important contribution to enhance our understanding of functional and dysfunctional coping strategies in the German general population and differences in well‐being during the COVID‐19 pandemic. Five latent coping profiles could be identified that differed in coping frequency and type of used coping strategies. Individuals using functional coping strategies were found to have significantly higher levels of well‐being than individuals who used primarily dysfunctional or low coping strategies. In turn, individuals with low or primarily dysfunctional coping reported an average well‐being score that was below the cut‐off score recommended by Topp et al. (2015) for the screening of clinically relevant depression. Thus, this study identified two coping profiles (Profile 4 and 5) that may be at high risk for depressive disorders and could benefit from a preventive training programme in the context of the COVID‐19 pandemic. With respect to the findings of this study, functional coping strategies should be specifically strengthened, whereas dysfunctional strategies need to be reduced. Pre‐pandemic randomized controlled trials have already indicated that resilience training (Steinhardt & Dolbier, 2008) or specific coping interventions (Chen et al., 2015) can increase functional coping strategies and improve well‐being. Similar results were reported in a prospective randomized intervention study on stress management training (SMT), which aimed to expand and balance coping profiles (Kaluza, 2000).

To investigate the causality of the relationship between coping profiles and well‐being, longitudinally studies are required. In addition, other concepts could be included, such as coping flexibility, which additionally considers coping repertoire, balance of coping profile, cross‐situational variability in coping, or fit between situation and coping (Cheng et al., 2014). To address the impact of pandemics on population well‐being, specific training programs should be evaluated to improve functional coping strategies during this and future pandemics that can enhance well‐being.

## CONFLICT OF INTEREST

No potential conflict of interest was reported by the authors.

## ETHICS STATEMENT

The study met all ethical regulations as required by the regulations of the ethics committees which was responsible for the respective study sites.

## INFORMED CONSENT

Informed consent to participate in the study was obtained from all participants.

## PERMISSIONS

Participants were informed that they were under no obligation to participate and that they could withdraw at any time from the study without consequences.

## Supporting information

Table S1

Table S2

## Data Availability

The detailed sociodemographic information of the dataset does not fully protect the anonymity of the respondents. For this reason, the entire dataset cannot be made publicly available. However, excerpts of the data on a higher aggregation level can be provided upon justified request by the last author.
