# Supplementary material for: Coping profiles and differences in well‐being during the COVID‐19 pandemic: A latent profile analysis
Source: Stress Health. 2022 Sep 16:10.1002/smi.3196. Online ahead of print. doi: 10.1002/smi.3196 (PMC9539043; doi:10.1002/smi.3196)
Supplement: Supplementary file 1 — Table S1 [file SMI-9999-0-s001.docx]

**Supplement 1**

**Table S1**

*Model results of the latent profile analysis*

|  | **Profile 1**  *High functional coping* | **Profile 2**  *Moderate functional coping* | **Profile 3**  *High functional & religious coping* | **Profile 4**  *Low coping* | **Profile 5**  *Moderate functional & dysfunctional coping* |
| --- | --- | --- | --- | --- | --- |
|  | ***M (SE)*** | ***M (SE)*** | ***M (SE)*** | ***M (SE)*** | ***M (SE)*** |
| **Brief-COPE** |  |  |  |  |  |
| Active Coping | 2.10 (0.06) | 1.41 (0.07) | 1.76 (0.07) | 0.66 (0.05) | 1.25 (0.07) |
| Planning | 2.25 (0.05) | 1.56 (0.07) | 2.00 (0.07) | 0.80 (0.07) | 1.87 (0.08) |
| Instrumental support | 1.68 (0.07) | 0.63 (0.04) | 1.30 (0.06) | 0.30 (0.04) | 1.39 (0.07) |
| Positive reinterpretation | 2.25 (0.07) | 1.77 (0.10) | 2.03 (0.08) | 0.81 (0.06) | 1.06 (0.05) |
| Acceptance | 2.12 (0.05) | 1.98 (0.05) | 2.01 (0.07) | 1.30 (0.06) | 1.25 (0.07) |
| Humour | 1.48 (0.06) | 1.24 (0.06) | 1.18 (0.07) | 0.77 (0.05) | 0.93 (0.07) |
| Religion | 0.30 (0.03) | 0.16 (0.02) | 2.23 (0.06) | 0.14 (0.02) | 0.26 (0.04) |
| Emotional support | 2.16 (0.03) | 1.15 (0.06) | 1.64 (0.07) | 0.56 (0.05) | 1.51 (0.08) |
| Self-distraction | 2.17 (0.05) | 1.68 (0.06) | 1.94 (0.07) | 0.91 (0.06) | 1.62 (0.07) |
| Denial | 0.27 (0.04) | 0.17 (0.02) | 0.29 (0.04) | 0.20 (0.03) | 0.97 (0.13) |
| Venting | 1.46 (0.10) | 0.74 (0.03) | 1.15 (0.06) | 0.49 (0.04) | 1.55 (0.07) |
| Substance use | 0.36 (0.04) | 0.26 (0.02) | 0.31 (0.05) | 0.31 (0.03) | 0.88 (0.12) |
| Behavioural disengagement | 0.41 (0.03) | 0.39 (0.02) | 0.49 (0.05) | 0.55 (0.04) | 0.85 (0.06) |
| Self-blame | 0.41 (0.05) | 0.18 (0.02) | 0.44 (0.05) | 0.15 (0.02) | 1.21 (0.17) |
| **Pandemic Coping Scale** |  |  |  |  |  |
| Healthy lifestyle | 2.03 (0.04) | 1.70 (0.06) | 1.96 (0.07) | 1.07 (0.05) | 1.24 (0.06) |
| Enjoyable activities | 2.33 (0.04) | 2.02 (0.05) | 2.12 (0.05) | 1.41 (0.05) | 1.46 (0.05) |
| Daily structure | 2.40 (0.05) | 2.10 (0.07) | 2.23 (0.07) | 1.35 (0.07) | 1.33 (0.07) |
| Preventive measures | 2.82 (0.02) | 2.79 (0.02) | 2.79 (0.03) | 2.45 (0.05) | 2.66 (0.04) |

*Notes.* Brief Cope, Pandemic Coping Scale: (0 = ‘I have not been doing this at all’; 1 = ‘I’ve been doing this a little bit’; 2 = ‘I’ve been doing this a medium amount’; 3 = ‘I’ve been doing this a lot’).
