# Supplementary material for: Coping profiles and differences in well‐being during the COVID‐19 pandemic: A latent profile analysis
Source: Stress Health. 2022 Sep 16:10.1002/smi.3196. Online ahead of print. doi: 10.1002/smi.3196 (PMC9539043; doi:10.1002/smi.3196)
Supplement: Supplementary file 2 — Table S2 [file SMI-9999-0-s002.docx]

**Supplement 2**

**Table S2**

*Multiple group analyses to examine differences in well-being between the coping profiles*

| **Chi-square tests** | *χ²* | *p* | *p*  *Bonferroni-*  *Holm adjusted* |
| --- | --- | --- | --- |
| Overall test | 503.68 | <.001^**^ | <.001^**^ |
| Profile 1 vs. 2 | 2.08 | .149 | .298 |
| Profile 1 vs. 3 | 4.95 | .026^*^ | .078 |
| Profile 1 vs. 4 | 82.21 | <.001^**^ | <.001^**^ |
| Profile 1 vs. 5 | 354.33 | <.001^**^ | <.001^**^ |
| Profile 2 vs. 3 | 1.72 | .189 | .298 |
| Profile 2 vs. 4 | 70.30 | <.001^**^ | <.001^**^ |
| Profile 2 vs. 5 | 397.71 | <.001^**^ | <.001^**^ |
| Profile 3 vs. 4 | 24.67 | <.001^**^ | <.001^**^ |
| Profile 3 vs. 5 | 181.57 | <.001^**^ | <.001^**^ |
| Profile 4 vs. 5 | 106.07 | <.001^**^ | <.001^**^ |

*Notes.* Profile 1: *High functional coping*. Profile 2: *Moderate functional coping*. Profile 3: *High functional & religious coping*. Profile 4: *Low coping*. Profile 5: *Moderate functional & dysfunctional coping*.
